# Association between different behavioral factors and dental caries among children attending the dental clinics in a sample from Saudi Arabia

**DOI:** 10.1186/s12903-023-02849-8

**Published:** 2023-04-02

**Authors:** Freah L. Alshammary, Amal A. Mobarki, Nadia F. Alrashidi, Ahmed A. Madfa

**Affiliations:** 1 Department of Preventive Dentistry, College of Dentistry, University of Ha’il, Ha’il, Kingdom of Saudi Arabia; 2 Present Address: General Dentist, Private Sector, Tabuk, Kingdom of Saudi Arabia; 3 Present Address: General Dentist, Private Sector, Ha’il, Kingdom of Saudi Arabia; 4 Present Address: Department of Restorative Dental Science, College of Dentistry, University of Ha’il, Ha’il, Kingdom of Saudi Arabia

**Keywords:** Behavioral factors, Children, Dental caries, Oral health, Prevalence, Socioeconomics, Saudi Arabia

## Abstract

**Background:**

This study aimed to assess the association between different behavioral factors and the prevalence of dental caries among children attending the dental clinic in a sample from the Hail and Tabuk regions, Saudi Arabia.

**Method:**

A cross-sectional study design was employed to determine the burden of dental caries in teeth and key associated factors among 6-12-year-old children who attended different dental clinics. The data was recruited from Hail and Tabuk districts, Saudi Arabia. The study included only Saudi nationals, whose parents could fill out the self-administered questionnaire and provide informed consent for their child’s dental examination at clinics. Children underwent a simple dental examination based on the World Health Organization diagnostic criteria for oral health surveys. The Decayed, Missed, Filled Tooth (DMFT) index developed by the World Health Organization (WHO) was utilized to assess dental caries. Descriptive statistics were performed to describe categorical variables. The mean DMFT was compared between girls’ and boys’ and the children from Hail and Tabuk regions using the Mann-Whitney U-test. The chi-square test was used to examine the association between different behavioral factors and the prevalence of dental caries.

**Results:**

Of the total 399 children examined, 203 (50.9%) were boys, whilst 196 (49.1%) were girls. The prevalence of dental caries was correlated with the cleaning tool, parental educational level, dental visits, and sugar consumption (*p* < 0.05). However, brushing frequency failed to demonstrate a correlation with the prevalence of dental caries (*p* > 0.05). The total mean DMFT for the studied sample was 7.81 (SD ± 1.9). Caries’ experience was made up mainly of decayed teeth. Decayed teeth made up an average of 3.30 (SD ± 1.07). The total mean of missing and filling teeth was 2.51 (SD ± 0.99) and 1.99 (SD ± 1.26) respectively. There was no statistically significant difference in the mean DMFT and gender or between Hail and Tabuk (*p* < 0.05).

**Conclusion:**

Saudi Arabia continues to have a high prevalence of dental caries compared to the global norm.

## Background

Social disparities in health are influenced by a variety of factors, including economic, cultural, and systemic characteristics of the healthcare system. Variations in living circumstances, education, socioeconomic standing, occupation, and social development are the root causes of health disparities and are linked to social variables [1]. Life expectancy, alcohol, and tobacco use, access to healthcare, mortality, and morbidity, and chronic diseases like diabetes are used as health indicators to measure these disparities [2]. Both the general and oral health of the population is significantly impacted by these differences.

Dental caries has long been a major issue in oral health [3]. It is detrimental to not just dental health but also general health and quality of life [4], especially in developing countries [5]. 60 to 90% of children have dental caries, according to the World Health Organization (WHO) [6]. All ages are affected by dental caries; however, children are more severely affected than adults. This multifactorial condition starts with demineralization of the enamel and can progress to a cavitated lesion that affects all of the tissues in the tooth, leading to tooth loss. This process results in trouble sleeping, discomfort when chewing, pain, and early tooth loss, all of which can have a negative impact on the quality of life of both children and teenagers [7, 8]. Despite the existence of excellent basic prevention interventions, dental caries continues to rank among the most common chronic diseases globally and is a significant burden on healthcare systems [9]. A part of the solution to this issue is to accurately assess the current load and make preparations for a full dental program.

According to the American Academy of Pediatric Dentistry (AAPD), early childhood caries (ECC) refers to any primary tooth in a child 71 months of age or younger that has one or more decaying (non-cavitated or cavitated lesions), missing (owing to caries), or filled tooth surfaces. ECC is a complicated, contagious, chronic condition [10]. Children’s quality of life and ability to function may suffer if ECC is left untreated since it can spread quickly and cause pain and oral infections [11].

The study of socioeconomic and cultural indicators of oral diseases, as well as procedures to assess their influence, have attracted a lot of attention in Saudi Arabia, the largest nation in the Middle East, over the past years [12, 13]. While attempts have been undertaken to assess Saudi Arabia’s oral health condition, since 1993, there has not been a consistently calibrated assessment of oral diseases in the Kingdom [14]. Saudi Arabia has a young population, with people under the age of 18 accounting for over 40% of the total. There is a well-established link between childhood dental caries and adult dental caries [15, 16]. Numerous local research [17–21] have demonstrated that the samples of children studied lacked proper dental hygiene and had high rates of caries. Furthermore, according to government data, while more than 80% of Saudis reside in cities, there are several rural regions spread across 2,250,000 square kilometers of the country, demanding a comparison between urban and rural centers [22].

Children in Saudi Arabia have a high incidence of dental caries, with a predictable frequency of about 80% [23]; other high-risk locations include the Middle East, Latin America, and South Asia [24]. To achieve optimal health, the World Health Organization (WHO) emphasizes the necessity of lowering the global burden of dental caries. In 2003, WHO and the World Dental Federation (FDI) established global oral health targets for 2020 to help planners and policymakers improve the state of oral health in their communities [25]. Saudi Arabia, along with other developing and semi-developed nations, is unfortunately limited in their ability to accomplish the WHO’s objectives due to information shortages regarding baseline information on oral health and population-particular primary changeable variables of dental caries. To effectively focus public health mitigation efforts, participating in activities in health care funding must be given a higher priority than the related challenges.

It is critical to collect baseline oral health data to prepare for and detect improvements in children’s oral health status. Several research on the prevalence of dental caries has been conducted in Saudi Arabia [12–14]. However, no studies have been done on dental caries among children in the Hail and Tabuk regions. Therefore, the present study aimed to assess the association between different behavioral factors and the prevalence of dental caries among children aged 6–12 years attending the dental clinic in a sample from the Hail and Tabuk regions, Saudi Arabia.

## Method

This cross-sectional study was used to assess the prevalence of dental caries in teeth and important related circumstances among children aged 6 to 12 who attended various dental clinics. This study was carried out between September 1, 2020, and August 15, 2021. The data was recruited from Hail and Tabuk districts, Saudi Arabia. Hail and Tabuk are cities located in North-Western Saudi Arabia. It has a population of more than 1,000,000.

All experiments were performed following relevant guidelines and regulations. The College of Dentistry at the University of Hail’s Ethical Committee approved this cross-sectional study (No.: H-2020-400). Also, informed consent was obtained from all their legal guardian(s). Before involving in questionnaire participation and dental examination, informed consent was asked from all their parents. Then, all of them had the right not to contribute to this research or to leave the current study before completion. The researcher explained the purpose of the research to all interviewees. Only Saudi nationals were included in this study, and only those whose parents could complete a self-administered questionnaire and provide their child’s dentist informed consent at a clinic.

The sample size was calculated using the Cochran’s formula for estimating sample size Eqs. [25, 26] as follows:

$$N= \frac{Z\alpha \times P (1-P)}{{D}^{2}}$$

Where: N: Minimum sample size; α is 0.05 and the critical value is 1.96); Zα: the critical value of the Normal distribution at α_/2_ (e.g., for a confidence level of 95%, and P: Prevalence of the outcome of interest (dental caries among children 65%, based on a recent Saudi study conducted among children aged 12 years in Riyadh; and D: Degree of precision.

The recommended sample size was 350 children. The sample was increased by approximately 10% to compensate for possible none or incomplete responses, thus it was 385 clients. The final sample size was 399 children. Non-probability convenience sampling technique was adopted to select the required sample size from attendees of different dental clinics.

### Clinical oral examination

According to the WHO diagnostic standards for oral health assessments, children received a straightforward dental examination [13]. A two, well-trained professional dentist performed the basic oral assessment of every child. An examination is performed whereas the kid lying in an exceedingly supine position 16 under daylight. They used during examination disposable gloves, mirrors, and probes after wiping off soft food debris from teeth with gauze. The dentists changed their role after every 10 children`s examinations to avoid their fatigue. This simple oral examination poses no harm to children. The Decayed, Missed, Filled Tooth (DMFT) index developed by the World Health Organization (WHO) was utilized to assess dental caries [35].

In a pre-study exercise, two examiners were involved in the calibration. The intra- and inter-examiner reliability was evaluated using a sample of 30 subjects. For both intra- and inter-examiner reliability, the kappa value was 0.84.

### Parental questionnaire

A self-administered structured Arabic questionnaire, adopted from WHO will be applied after clinical oral examination to define demographic (gender, residency, region, and educational level of parents) and behavioral characteristics (frequency of cleaning teeth, tooth cleaning device, use of toothpaste, frequency of sugar consumption, previous dental visits, and their reasons) of the participants.

### Statistical analysis

IBM applied math Package for Social Sciences (SPSS) for Windows, Version 25.0 (IBM Corp., Armonk, NY) software was utilized for data analysis. Descriptive statistics (e.g., frequency, percentage) was performed for categorical variables whereas mean and standard deviation were utilized to describe categorical variables. The chi-square test was used to examine the association between different behavioral factors and the prevalence of dental caries. The mean DMFT was compared between girls’ and boys’ children and the children from Hail and Tabuk regions using the Mann-Whitney U-test. Probability values of *p* < 0.05 were considered to be statistically significant.

## Results

Table 1 shows the demographic and oral health determinants of the participants. Of the total 399 children examined, the percentage of boys and girls examined was 203 (50.9%) and 196% (49.1), respectively. Most participants are from town 373 (93.5%). The study revealed that 199 (49.9%) of the participants were from Hail and 200 (50.1%) were from Tabuk. About 155 (38.8%) and 133 (33.3%) of the participant’s parents had university and secondary education, respectively. However, the children that had primary school-educated parents were 111 (27.8%). The study revealed that 46.6% and 30.9% of the participants brushed their teeth once per day or less respectively. However, the children that brushed their teeth twice per day were 22.6%. most of the participants used toothbrushes 95%. Most children used toothpaste during brushing their teeth 87.5%. About 57.9% of the participants always consume sugar and 41.4% of children are sometimes taken sugar. About 88.2% of the participants had visited the dentist before, while the survey revealed that 45.1% visited clinics due to dental pain and 22.3% for treatment. About 15.3% of the participants visited clinics for a checkup.

**Table 1:** Associations between the demographic and the oral health determinants and the prevalence of dental caries

Variable		Frequency n (%)	Dental Caries		P-value
			Yes	No	
**Gender**	Boys	203 (50.9)	157 (39.3%)	46 (11.5%)	0.069
	Girls	196 (49.1)	145 (36.3%	51 (12.8%	
**Residency**	Town	373 (93.5)	281 (70.4%)	92 (23.1%)	0.164
	village	26 (6.5)	21 (5.3%)	5 (1.3%)	
**Region**	Hail	199 (49.9)	149 (37.3%)	50 (12.5%)	0.086
	Tabuk	200 (50.1)	153 (38.3%)	47 (11.8%)	
**Educational level of parents**	Primary	111 (27.8)	80 (20.1%)	32 (7.8%)	0.050
	Secondary	133 (33.3)	105 (26.3%)	28 (7.0%)	
	University	155 (38.8)	117 (29.3%)	38 (9.5%)	
**Brushing frequency**	Sometimes	123 (30.8)	90 (22.6%)	33 (8.3%)	0.061
	Once a day	186 (46.6)	145 (36.3%)	41 (10.3%)	
	Twice a day	90 (22.6)	67 (16.8%)	23 (5.8%)	
**Tool of cleaning**	No	10 (2.5)	6 (1.5%)	4 (1.0%)	0.036
	Miswak	9 (2.3)	6 (1.5%)	3 (0.8%)	
	Toothpick	1 (0.3)	1 (0.3%)	0 (0%)	
	Toothbrush	379 (95.0)	289 (72.4%)	90 (22.6%)	
**Toothpaste**	Yes	349 (87.5)	263 (65.9%)	86 (21.6%)	0.132
	No	50 (12.5)	39 (9.8%)	11 (2.8%)	
**Sugar consumption**	Never	3 (0.8)	3 (0.8%)	0 (0%)	0.050
	Sometimes	165 (41.4)	119 (29.8%)	46 (11.5%)	
	Always	231 (57.9)	180 (45.1%)	51 (12.8%)	
**Dental visits**	Yes	352 (88.2)	262 (65.7%)	90 (22.6%)	0.041
	No	47 (11.8)	40 (10.0%)	7 (1.8%)	
**If yes**	Tooth pain	180 (45.1)	137 (34.3%)	42 (10.5%)	0.035
	Check-up	61 (15.3)	43 (10.8%)	18 (4.5%)	
	Treatment	89 (22.3)	67 (16.8%)	22 (5.5%)	
	I don’t remember	69 (17.3)	55 (13.8%)	14 (3.5%)	

Table 1 shows the association between different behavioral factors and the prevalence of dental caries among children attending the dental clinic. Tool of cleaning, educational level of parents, dental visits, and the consumption of sugar were associated with the prevalence of dental carious (*p* < 0.05). However, no association was found between gender, residency, region, and the prevalence of dental caries (*p* > 0.05). Moreover, brushing frequency did not show an association with the prevalence of dental caries (*p* > 0.05).

Table 2 shows the mean and standard deviation DMFT values of the study sample. The total mean DMFT for the boys and girls were 7.84 (SD ± 1.93) and 7.80 (SD ± 1.87), respectively. For region, the total mean DMFT for Hail was 7.70 (SD ± 1.84), however, for Tabuk was 7.94 (SD ± 1.95). The total mean DMFT for the studied sample was 7.81 (SD ± 1.90). Caries experience was made up largely of decayed teeth. Decayed teeth made up an average of 3.30 (SD ± 1.07). The total mean of missing and filling teeth was 2.51 (SD ± 0.99) and 1.99 (SD ± 1.26) respectively. Using the Mann-Whitney U-test, there was no statistically significant difference between the mean DMFT and gender (*p* > 0.05) as displayed in Table 3. Additionally, Mann-Whitney U-test found that there were not statistically significant between the mean DMFT and region (*p* < 0.05) as shown in Table 3.

**Table 2:** Mean and standard deviation DMFT values of the study sample

Variable		Dental decay	Missing	Filling	DMFT
**Gender**	Boys Mean (SD)	3.2364 (1.05457)	2.4926 (1.02140)	2.1083 (1.26572)	7.8374 (1.93921)
	Girls Mean (SD)	3.3826 (1.10081)	2.5408 (0.97316)	1.8775 (1.25485)	7.8010 (1.86635)
**Region**	Hail Mean (SD)	3.2261 (1.10742)	2.5326 (0.93078)	1.9447 (1.27206)	7.7035 (1.84706)
	Tabuk Mean (SD)	3.3900 (1.04564)	2.5000 (1.06095)	2.0450 (1.06095)	7.9350 (1.95187)
**Study sample**	Mean (SD)	3.3083 (1.07867)	2.5163 (0.99704)	1.9950 (1.26411)	7.8195 (1.90147)

**Table 3:** Association between the gender and the region and the DMFT

Variables		N	Mean Rank	Sum of Ranks	P-value
**Gender**	Boys	203	200.85	40772.50	0.175
	Girls	196	199.12	39027.50	
**Region**	Hail	199	192.36	38279.50	0.182
	Tabuk	200	207.60	41520.50	

Mann-Whitney U test shows no statistically significant difference between either boys and girls or between children from Hail and Tabuk (p > 0.05)

## Discussion

Dental caries is one of the main public health problems in Saudi Arabia [14]. Despite the rise in Saudi Arabian literature on dental caries, the last calibrated survey of the entire region was done more than 20 years ago [28]. Aljanakh et al. [29] did one study in Hail City. They selected high school between the ages of 16 and 18 to represent their sample. The prevalence of dental caries among children in the Hail and Tabuk regions, however, has not been studied. Therefore, the current study aimed to determine the relationship between several behavioral characteristics and the frequency of dental caries among children aged 6 to 12 who visited the dental clinic in a sample from the Hail and Tabuk regions, Saudi Arabia.

This study demonstrates the importance of parental factors, such as educational level, oral health practices, access to healthcare services, and other characteristics, which are related to children’s dental health. This supported the recent study [27,30].

Boys and girls have different oral health statuses due to cultural and socioeconomic inequalities that affect how exposed they are to risk factors and how easily they can receive preventive factors and care. There are associations between gender and dental caries, despite the possibility that girls should have a higher caries rate due to earlier tooth emergence and longer exposure to cariogenic processes. Our research found no significant difference between boys and girls. Studies conducted by Popoola and Denloye [31] and Khan [32] revealed similar outcomes.

Even though education and income are progressively more linked to oral health, one of the greatest significant factors in research analyzing health disparities in children is the degree of learning of the parents. This is due to the increased likelihood of bad oral health in terms of dental caries in people who have low incomes and low levels of education. In a study of 6-year-old children, Van der Tas et al. [33] observed a connection between dental caries and low income and highlighted that the level of education of the mother was a crucial indicator for the prevalence of dental caries. A meta-analysis by Schwendicke et al. [34] found that a poor income was linked to a higher risk of developing caries lesions and that the existence of a minimal learning level enhanced the likelihood of developing caries. Similarly, parental education affects family income, career prospects, and involvement in social safety programs [35]. As a result, it is discovered that these variables affect how easily people can obtain health care and that relatives with lower learning attainment are probable to experience negative well-being consequences.

According to the results of the current study, sugar consumption was linked to the advancement of tooth decay lesions in the survey group, proving that it has a significant impact on how caries develop. According to studies by Quadri et al. [36] on participants aged 6 to 15 years old, those whose moms had less education as well as those who consumed more sugary foods were more likely to have caries. Therefore, prevention strategies and education about eating habits and dental health should focus on mothers of young children to avoid the spread of caries. To avoid the detrimental consequences of these habits on general health, it is also crucial to develop appropriate sugar and beverage consumption practices from an early age [37].

Lack of brushing encourages the buildup of biofilm on all dental surfaces, which leads to the emergence of caries lesions. Different events that happen at the individual, family, and community levels may be the cause of the association between oral hygiene and socioeconomic status [38]. Furthermore, socioeconomic status as well as poor dental hygiene may have a detrimental effect on quality-of-life children [39]. There is evidence that toothpaste use when brushing is a prospective justification for the lower occurrence of tooth decay, in addition to the fact that toothpaste is an efficient agent of remineralization, as shown by the current study’s result that over 90% of participants used toothpaste [40]. Given the high incidence of caries and the fact that most pupils use toothpaste, it is reasonable to assume that additional socioeconomic factors are contributing to the rise in caries prevalence in this demographic.

The current study discovered that 11.8% of the participants had not been to the dentist and that those participants who reported fewer dental visits had more caries lesions than their counterparts who attended the dentist more frequently. Generally, participants from low-income households had fewer dental visits than those from high-income. Installing a dentist’s office in a primary school is one way to provide dental care to students from low-income households. This is conducted to improve dental service usage and lower caries prevalence. Additionally, it is crucial to put new ideas into practice that increase household income and eliminate health inequities to reduce socioeconomic determinants of health [41].

The prevalence of dental caries in the present research was 76%. Our sample of primary school students had a significant prevalence of dental caries, which was comparable with other research from Saudi Arabia [42–46] and the United Arab Emirates [47]. The prevalence was shown to be 80% in the latest meta-analysis of numerous dental caries studies conducted in diverse Saudi Arabian provinces [4]. Furthermore, the observed prevalence of dental caries among youngsters in the current study was much greater than the WHO/FDI target set for the year 2000 (50%) [48]. A burden on public health, dental caries is endemic in the Middle Eastern population, according to the findings from our study and other studies.

A good predictor of the severity of a dental caries pandemic is the prevalence of dental caries, or the proportion of children with at least one dental caries lesion. Overall, the results of this study are consistent with recent studies in the Kingdom [43, 49, 50]. Moreover, the current study agrees with Wyne [14], who reported that the overall caries prevalence among the sample from Riyadh, Saudi Arabia was 74.8%. Although the availability of preventive and therapeutic dental services has increased significantly throughout Saudi Arabia over the past years, it is outside the purview of this study to determine whether these services were actually used or whether they contributed to the decline in dental caries [50]. The level of dental caries revealed in the present investigation is consistent with the latest database findings from Saudi Arabia and the United Arab Emirates [15, 50].

Given that the majority of comparable studies have employed the DMFT index, some of the limitations of the present study are related to its cross-sectional design and comparison with another research. The understanding of the sociodemographic disparities in dental caries would improve as a result of longitudinal designs. Lastly, because different research employs different markers to assess socioeconomic class, this could make the present study less comparable.

## Conclusions

Under the limitation of the current research, it can be concluded that Saudi Arabia continues to have a high prevalence of dental caries when compared to the global norm. Dentistry professional officials in Saudi Arabia should pay quick attention to the critical dental public health issue of childhood caries. It is impossible to judge whether oral health objectives are being met without the capacity to describe the existing situation. To improve the oral health of Saudi Arabian children, we urgently need a blueprint with a clear beginning, end, and pathway. To come up with effective caries planning plans for the Kingdom, further data analysis is required, focusing on sociodemographic characteristics.

## Data Availability

The datasets generated and/or analyzed during the present study are not publicly available as ethics approval was granted on the basis that only the researchers involved in the study could access the identified data but are available and accessible from the corresponding author upon reasonable request.

## Abbreviations

ECC: Early childhood caries
AAPD: American Academy of Pediatric Dentistry
SD: Standard Deviation
WHO: World Health Organization
FDI: Fédération Dentaire Internationale
